# Case Report: Serous borderline ovarian tumor and extensive abdominopelvic endometriosis mimicking advanced epithelial ovarian cancer in a postmenopausal patient

**DOI:** 10.3389/fmed.2025.1581241

**Published:** 2025-06-17

**Authors:** Katherine Livatova, Anthony D. Nguyen, John Pizzuti, Yi-Chun Lee, Jennifer McEachron

**Affiliations:** ^1^Department of Obstetrics and Gynecology, Good Samaritan University Hospital, West Islip, NY, United States; ^2^Department of Anesthesiology, Good Samaritan University Hospital, West Islip, NY, United States; ^3^Division of Gynecologic Oncology, Good Samaritan University Hospital, West Islip, NY, United States

**Keywords:** case report, endometriosis, serous borderline ovarian tumor, exploratory laparotomy, postmenopausal

## Abstract

The lifetime risk of ovarian cancer is 1 in 75, and it is often diagnosed at an advanced stage due to the lack of effective screening. Epithelial ovarian cancer (EOC) is the most common type of ovarian cancer in older patients, with borderline ovarian tumors (BOTs) accounting for 15% of EOC cases. BOTs are usually of low malignant potential. Since platinum-based chemotherapy has not been shown to improve survival rates, cytoreductive surgery (CRS) is recommended as a treatment option. Additionally, endometriosis affects 10.5% of patients with EOC. It often progresses to clear cell and endometrioid ovarian cancer. This progression is believed to result from repetitive cycles of inflammation, mutations in multiple pathways, and microsatellite instability. While other types of cancers, such as BOTs, can arise in cases of endometriosis, they are less common. This case report describes an unusual case of a 62-year-old female patient who appeared to present with advanced-stage epithelial ovarian cancer (EOC) but was unexpectedly diagnosed with a borderline ovarian tumor (BOT) accompanied by extensive endometriosis. The patient’s history of endometriosis, infertility, and low parity increased her risk of developing a BOT; however, her age at presentation was unusual. The key clinical feature of this case that contributes to the existing literature is the vital role of pathology in guiding chemotherapy decisions, particularly when there is disease progression despite ongoing treatment. This highlights the necessity of a thorough history and physical examination, as several aspects in the patient’s medical history were suggestive of a BOT. The patient initially presented with emesis, abdominal distention, postmenopausal bleeding, and a large, rapidly growing pelvic mass, which raised the initial suspicion of advanced EOC. However, she was unable to undergo surgery due to supraventricular tachycardia and venous thromboembolism (VTE) caused by the mass effect of the tumor. Despite being medically unstable, an exploratory laparotomy with resection of the mass was performed. Pathology revealed a stage 1C3 serous BOT within a large endometrioma. Chemotherapy was not required postoperatively. The patient remained under close observation by gynecologic oncology for surveillance, hematology for managing her VTE, and cardiology for monitoring her supraventricular tachycardia (SVT).

## Introduction

A woman’s lifetime risk of developing ovarian cancer is 1 in 75, with the incidence increasing with age (1, 2). Due to the lack of effective screening, many cases are diagnosed only after the cancer has spread beyond the pelvis, classifying it as an advanced stage. Advanced EOC carries a high risk of recurrence and poor long-term survival outcomes, with a 5-year survival rate of 50% for patients diagnosed with stage III and IV disease (3). Advanced ovarian cancer most often presents as abdominal pain or pressure (44%), general abdominal swelling without a detectable mass (39%), and GI symptoms (15%) (3). The incidence of EOC is higher in individuals with endometriosis, a non-invasive disease affecting 10–15% of female individuals of reproductive age. This increased risk is believed to arise from similarities in molecular alterations associated with both conditions (4). Patients with endometriosis may also have symptoms such as pelvic discomfort, malaise, and gastric and urinary symptoms (5). Despite the prevalence of endometriosis, fewer than 1% of patients develop ovarian malignancy. These patients usually develop endometrioid or clear cell carcinomas, but endometriosis can exist on a spectrum with BOTs (1, 2, 6, 7). BOTs are a distinct entity within the spectrum of EOC, characterized by their cytologic atypia without stromal invasion (1, 4). These tumors present with symptoms similar to advanced EOC, but up to 30% report no symptoms (8). BOTs are treated with cytoreductive surgery (CRS) alone compared to the combination of CRS and chemotherapy for advanced EOC (8, 9). In this report, we present a case of suspected advanced ovarian malignancy in a patient with high medical risk for surgery, who experienced disease progression despite chemotherapy, ultimately being diagnosed with borderline pathology.

## Case presentation

A 62-year-old woman presented to the emergency department (ED) with abdominal distention, hematemesis, and postmenopausal bleeding. She reported experiencing abdominal distention over the past 1–3 months, which had worsened acutely in the last week. In addition, she was unable to tolerate oral intake for the past 12 h due to intractable emesis. Although she had not seen an gynecologist in 10 years, she reported a history of endometriosis and large ovarian cysts, for which she had declined surgical intervention 20 years ago. She also described a 10-year history of infertility, despite undergoing multiple treatments, leading to the adoption of her only child.

**Figure 1:**
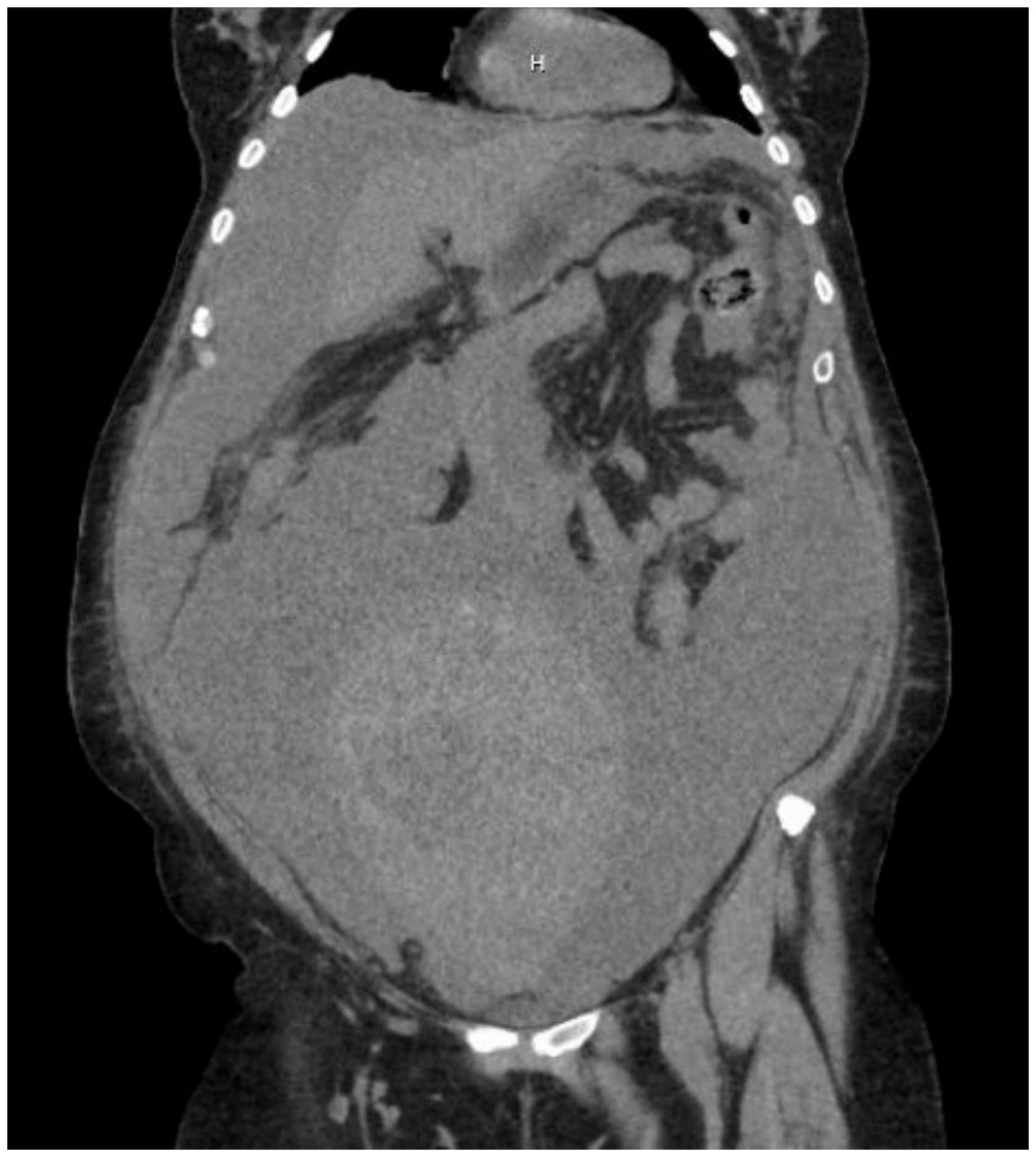
CTAP without contrast showing a large, heterogenous, lobulated pelvic mass measuring 12 × 2.4 × 17.2 cm, with soft tissue and cystic components, along with a smaller deep pelvic mass measuring 5.7 cm.

In the emergency department (ED), she was tachycardic and hypotensive with normal oxygen saturation. A computed tomography scan of the abdomen and pelvis (CTAP) revealed a large, heterogenous, lobulated pelvic mass measuring 12 × 2.4 × 17.2 cm, with both soft tissue and cystic components. In addition, a smaller deep pelvic mass measuring 5.7 cm was identified, as shown in Figure 1. There was a left adnexal cystic structure measuring 5.5 × 3.4 cm and a large amount of ascites. Interventional radiology drained 5.0 L of turbid brown fluid, but noted a moderately large amount of ascites remaining. During her hospitalization, she underwent an additional paracentesis, draining another 5.1 L of ascites. Her CA125 level was 7,849 and CEA level was 25, resulting in a ratio of 314, which suggested a malignancy of ovarian origin. She was medically optimized and discharged on hospital day 4, with plans for outpatient diagnostic laparoscopy and possible CRS for suspected advanced ovarian cancer.

The patient returned to the hospital 1 week later for her scheduled surgery. Upon induction of anesthesia, her blood pressure dropped, and she developed supraventricular tachycardia (SVT) with a heart rate ranging from 190 to 220 beats per min. She was deemed too unstable for surgery, and the procedure was aborted. Paracentesis was performed instead, with 1.2 L of fluid removed. She was converted out of SVT, and further cardiac workup was negative.

Despite high clinical suspicion for ovarian cancer, peritoneal cytology was found to be negative for carcinoma. However, based on imaging findings and the CA125/CEA ratio, a multidisciplinary tumor board recommended the initiation of paclitaxel, carboplatin, and bevacizumab chemotherapy for NACT for presumed advanced EOC. The initiation of chemotherapy was delayed due to symptomatic anemia and the need for multiple transfusions. She received her first chemotherapy cycle approximately 4 weeks after her initial presentation.

Between cycles 1 and 2, she returned to the ED with symptomatic ascites and tachycardia. She was found to be in SVT with a saddle pulmonary embolism on CT angiogram, along with echocardiographic evidence of right heart strain. There was significant venous thromboembolism affecting the inferior vena cava at the level of the renal vessels, spreading distally to the common iliac vessels. The patient was admitted to the medical ICU, where she was converted to sinus rhythm and managed with an IV heparin drip. An Inari procedure, which involves passing a catheter through the femoral vasculature to remove the clot mechanically, was attempted but could not be performed due to occlusion of the IVC secondary to an enlarging abdominopelvic mass. A repeat CT scan showed that the dimensions of the mass had increased to 28 × 17 × 27 cm, reflecting a 10 cm increase in size. The patient was medically optimized and discharged for outpatient management.

**Figure 2:**
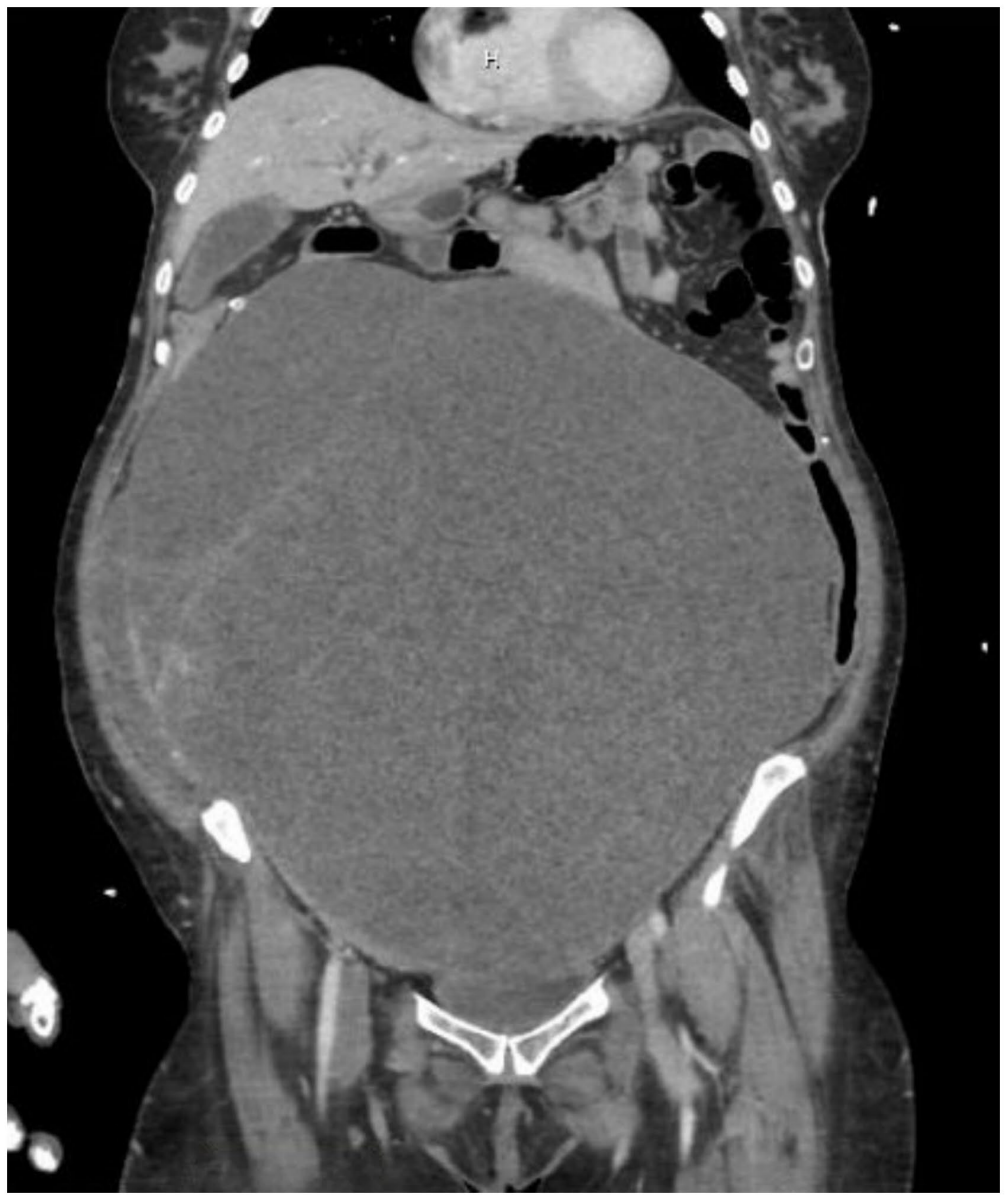
CTAP showing the pelvic mass now measuring 30.2 × 16.8 × 31.1 cm, compared to the original size of 17.2 cm. Imaging also demonstrated a stable, large clot burden involving the vena cava and common iliac vessels.

She resumed chemotherapy but returned to the ED after cycle 3 for syncope. She was found to have neutropenia and *C. difficile* infection. Repeat CTAP, as shown in Figure 2, showed that the pelvic mass had increased to 30.2 × 16.8 × 31.1 cm, compared to the original size of 17.2 cm. Imaging also demonstrated a stable, large clot burden involving the vena cava and common iliac vessels. During this admission, a multidisciplinary team, which included specialists from gynecologic oncology, cardiology, anesthesiology, vascular surgery, and cardiac surgery, held a meeting to develop a management plan and consider offering the patient surgery despite the high cardiac risk involved. The decision to proceed with surgery was made due to the lack of response to chemotherapy, worsening functional status, a suspected isolated abdominal mass, and the absence of a confirmatory diagnosis. All possible intra-operative outcomes were considered, including the potential risk of major cardiopulmonary compromise in the event of a dislodged venous clot, which could worsen embolic burden on the right ventricle, leading to hemodynamic collapse. The patient and family ultimately decided to proceed with high-risk debulking surgery, understanding her poor status and the associated morbidity risk.

She underwent an exploratory laparotomy, total abdominal hysterectomy, bilateral salpingo-oophorectomy, omentectomy, resection of the pelvic mass, ureteroneocystostomy, right double-J ureteral stent placement, appendectomy, and lysis of adhesions on 11/27/2023. Prior to the induction of anesthesia, an arterial line and central venous catheter were placed. During the induction of anesthesia and throughout the procedure, an experienced anesthesia team monitored the patient with a continuous echocardiogram to identify and manage any strain on the right ventricle resulting from new clot burden or fluid shifts. Intraoperatively, there was a 35 cm ovarian mass causing mass effect on the inferior vena cava, aorta, and other retroperitoneal structures. Approximately 8.5 L of hemorrhagic fluid was removed, and a 6 cm solid component was noted within the mass. There was evidence of spillage into the abdomen that had occurred preoperatively. Deep infiltrating endometriosis and adhesions were noted throughout the abdominopelvic cavity, which included powder-burn lesions and clear vesicles involving multiple organs. A frozen section was sent at the beginning of the case and promptly returned with a diagnosis of borderline pathology. Due to this diagnosis and prior ascites testing being negative, the decision was made not to obtain peritoneal washings. During dissection of the pelvic sidewalls to identify vital structures, some powder-burn lesions and vesicular lesions were removed along with the specimen. Extensive resection of endometriosis was avoided to minimize operative time due to the high-risk nature of the procedure and the severe comorbidities of the patient. There was complete gross resection of the mass (R0) and extensive pelvic sidewall dissection. After removal of the mass, it was found that the right ureter had been kinked and devascularized from the weight of the mass, requiring resection and ureteroneocystostomy. She was transferred to the surgical ICU postoperatively and received multiple transfusions of various blood products. Her hospital course was prolonged due to preoperative deconditioning. She was discharged to a subacute rehab on postoperative day 30.

**Figure 3:**
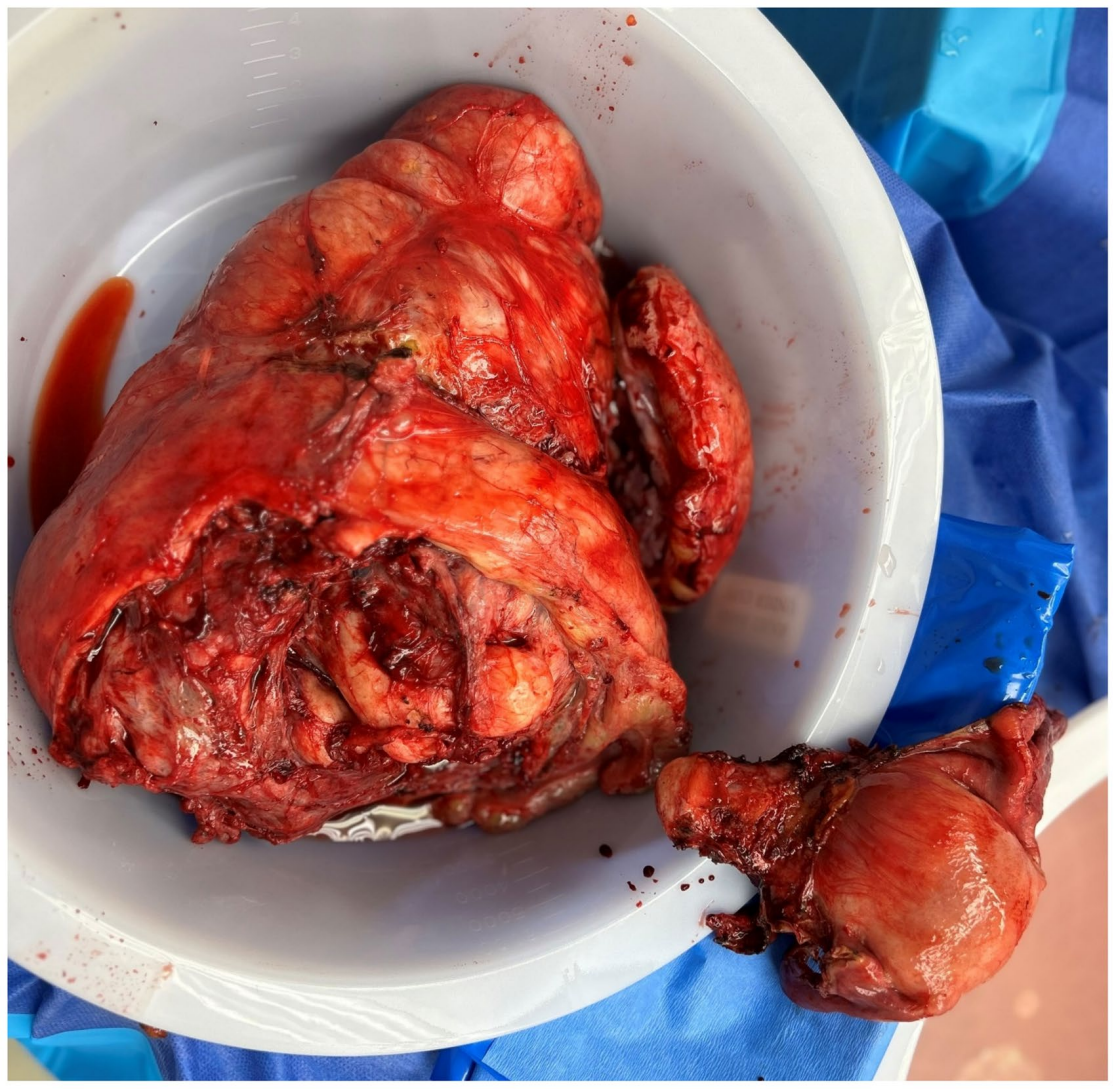
Gross specimen of the uterus, tumor, and endometrioma after removal.

Final pathology confirmed a stage IC3 serous borderline tumor. Therefore, there were no invasive implants, and chemotherapy was not indicated. The tumor was 5 cm in size and discovered within a 22 cm denuded hemorrhagic cyst with abundant hemosiderin deposit, fibrosis, and hemorrhage—features consistent with an endometriotic cyst. The specimen also showed extensive adhesions, endometrial glands and stroma, and large bundles of smooth muscle and fibroadipose tissue, as shown in Figure 3. Her CA125 trend is provided in Table 1. Despite her CA125 levels decreasing with neoadjuvant chemotherapy, the size of the mass and its mass effect did not improve. Withholding chemotherapy due to the patient’s medical instability caused the CA125 levels to rise again, and only after complete gross surgical resection did the CA125 levels return to normal levels. Since discharge, the patient has followed up closely with her gynecologic oncologist. A 6-month post-operative CT of the chest, abdomen, and pelvis was performed, which showed no evidence of the disease. She also followed up with her cardiologist, who performed a cardiac stress test and started her on metoprolol, as well as with her hematologist, who confirmed the resolution of her VTE. She no longer requires anticoagulation.

**Table 1:** Dates and values of the patient’s CA125 tumor marker levels throughout the course of her multiple hospitalizations.

Date	CA125
8/4/23	7,849
9/6/23	402
10/4/23	309
11/1/23	1,451
9/9/24	9

The CEA level was only measured once during the initial presentation.

CEA negative.

## Discussion

In this report, we describe a case of suspected advanced EOC, managed with chemotherapy, which was ultimately diagnosed as a serous borderline tumor in the setting of endometriosis, occurring in an uncharacteristically older age group. BOTs have low malignant potential and a 10-year survival rate of 95%. They account for 15% of EOC cases, with 50% of these cases being mucinous and 44% being serous (8). The first-line treatment is surgical resection. Adjuvant platinum-based chemotherapy has no beneficial effect on survival rates (6). BOTs tend to occur in younger women, with one study finding that 82% of cases occurred in women under the age of 60 (3). Another study found that endometriosis-associated BOTs tend to occur in premenopausal women, with an average age of 44 years (10).

Endometriosis exists on a spectrum with EOC. Many cases are benign; however, the repetitive cycle of inflammation can lead to the production of sex steroid hormones and a microenvironment that allows for malignant transformation (7). This can lead to atypical endometriosis, which is characterized by cytologic atypia and hyperplasia on histological examination and is more likely to progress to BOTs. It has been shown that women with a long-term history of endometriosis, endometriomas measuring greater than 9 cm, and proven atypical endometriosis have a higher risk of progression to EOC and should be monitored closely (11). Epithelial cancers associated with endometriosis tend to occur in younger patients and share symptoms with endometriosis, making it difficult to distinguish between the two in a clinical setting. Patients with BOTs tend to have non-specific symptoms, such as abdominal pain, vaginal bleeding, and sexual discomfort (6), which are symptoms commonly reported in endometriosis. BOTs also tend to lack the typical malignant ultrasound findings of ovarian cancer, making them more difficult to distinguish from benign masses. Endometriosis progresses to ovarian cancer in 0.3–0.8% of cases. The risk further increases in women over the age of 50 or those with a history of endometriosis lasting more than 10 years (7, 11).

Endometriosis has been found in 10.5% of patients with BOTs or invasive EOC. These patients are usually younger, with lower parity, and premenopausal (7). Endometriosis has malignant potential due to mutations in the AR1D1A gene and high estrogen levels, leading to the malignant transformation of endometriotic cysts (10). Endometriosis most commonly progresses to Type 1 tumors, which are characterized by mutations in K-RAS, B-RAF, PTEN, beta catenin/Wnt, and microsatellite instability (7). These tumors are more likely to be of the clear cell or endometrioid carcinoma type (5). BRAF or KRAS mutations are found in 88% of BOTs and are believed to be early mutations in their development (6). Endometriosis rarely progresses to Type II tumors, which include high-grade serous or undifferentiated carcinomas, and is dependent on p53 mutations (7). P53 mutations contribute to the malignant transformation of endometriosis and increase the likelihood of invasiveness in serous BOTs (6). This is due to the decreased expression of E-cadherin, which occurs because of the activation of the PI3K/AKT pathway (4). Fortunately, it has been shown that ovarian cancer associated with endometriosis, despite occurring in a younger population, has a better prognosis, is more likely to be diagnosed at an earlier stage, and has a lower tumor grade compared to spontaneously occurring EOC (1).

The patient’s history of infertility, despite treatment, is associated with a diagnosis of endometriosis and increased her risk of serous BOTs. A nationwide study found that the odds ratio for a serous BOT in the setting of infertility was 3.31, with a significant decrease in risk as parity increased (12).

At initial presentation, due to her cardiac contraindications to surgery, the decision was made to proceed with NACT based on the clinical picture and elevated CA125/CEA ratio. This ratio has been used in clinical trials to differentiate primary ovarian tumors from those originating in other primary sites, such as the gastrointestinal tract. It has been demonstrated that a CA125/CEA ratio of 25 or lower requires colonoscopy, gastroscopy, and mammography to be performed to rule out the possibility that the pelvic mass is related to metastatic cancer from another source (9). Our patients’ CA125/CEA ratio was greater than 300, supporting a diagnosis of primary ovarian malignancy. Endometriosis is known to cause elevations in CA125, with case series reporting a wide range of values (13–15). BOT presentation in this age group is unusual, especially with her concomitant diagnosis of endometriosis. The lack of response to chemotherapy is characteristic of BOTs. The patient had several additional risk factors for a serous BOT, including endometriosis, low parity, and infertility.

There are case reports describing patients with giant borderline ovarian tumors, defined as those measuring at least 10 cm in diameter (16, 17). One case involved a 30 cm infected borderline ovarian tumor; however, the patient was stable and around 30 years old (16). Her younger age contributed to her improved stability and ability to safely undergo surgery despite the large size of her tumor. Other case reports describe giant borderline ovarian tumors that were either smaller than the one in this case or occurred in younger patients, allowing them to be more stable during surgery (18, 19). A case of a 74-year-old female with a 36 cm tumor has also been reported; however, she was stabilized with a few units of packed red blood cells, and her intraoperative volume status was managed by slowly draining the tumor while replacing fluids intravenously (17). Unfortunately, this was not possible in our case due to our patient decompensating immediately upon induction of anesthesia. This case is unique due to the patient’s inability to tolerate surgery and her postmenopausal age, and it highlights the importance of multidisciplinary cooperation and continuous monitoring in the management of complex ovarian cancer cases that require surgical intervention.

Due to the patient’s instability, definitive treatment was delayed, and medical decompensation occurred. Although this patient never underwent MRI, studies have shown that BOTs share MRI features with other benign and malignant diseases, and definitive diagnosis based on MRI findings is difficult (20). An IR biopsy of the mass could also be considered for a definitive diagnosis. However, even with a definitive diagnosis, the patient’s medical stability would not have improved. A multidisciplinary meeting would still have been necessary, and the surgery would have still carried a high risk of morbidity and mortality. Holding a multidisciplinary meeting as early as possible in the setting of failed chemotherapy would likely benefit other similar patients in the future.

A key strength of this case is that the patient received continuous care within the same hospital and its affiliated subspecialties, so there is complete access to her medical records. In addition, the institution had all the necessary resources to perform the high-risk surgery on-site, eliminating the need for transfer to another facility. A limitation of this case is that it was not possible to obtain the patient’s medical records prior to her initial presentation. The key clinical insight that this case adds to the existing literature is the critical role of pathology in guiding chemotherapy decisions, especially in situations where there is disease progression despite chemotherapy. It highlights the essential role of a thorough medical history and physical examination. The patient’s distant history, dating back to her early 20s and including severe endometriosis and infertility treatments, was critical in assessing her risk for developing future cancers and understanding the potential impact of chronic, long-term endometriosis on her current imaging and laboratory findings. Collaboration between the surgical team and anesthesia was critical in achieving a successful outcome and minimizing the risks of cardiopulmonary collapse. Currently, the patient is alive, with no evidence of disease, and no longer requires long-term anticoagulation.

## Data Availability

The original contributions presented in the study are included in the article/Supplementary material, further inquiries can be directed to the corresponding author.
