# Supplementary material for: Case Report: Serous borderline ovarian tumor and extensive abdominopelvic endometriosis mimicking advanced epithelial ovarian cancer in a postmenopausal patient
Source: Front Med (Lausanne). 2025 Jun 17;12:1581241. doi: 10.3389/fmed.2025.1581241 (PMC12209280; doi:10.3389/fmed.2025.1581241)
Supplement: Supplementary file 1 [file Table_1.docx]

| Date | Event |
| --- | --- |
| 8/4/23 | Pt presented to emergency room- imaging demonstrates large pelvic mass, admitted for hemodynamic instability. CA125- 7849 |
| 8/10/23 | Pt discharged after drainage of a total of 10.1L ascites. |
| 8/15/23 | Pt presents for diagnostic laparoscopy but was aborted due to SVT and hemodynamic instability. Additional paracentesis performed. |
| 8/18/23 | Pleurx placed and patient discharged from hospital. |
| 9/6/23 | First cycle of paclitaxel, carboplatin, bevacizumab. |
| 9/19/23 | Patient admitted and diagnosed with saddle PE and extensive VTE, started on anticoagulation. |
| 10/4/23 | Second cycle of paclitaxel, carboplatin, bevacizumab. |
| 11/1/23 | Third cycle of paclitaxel, carboplatin, bevacizumab. CA125 of 1451. |
| 11/27/23 | Exploratory Laparotomy, Total Abdominal Hysterectomy Bilateral Salpingoophorectomy, Omentectomy, Resection of Pelvic Mass, Tumor Debulking, Bilateral Ureterolysis, Cystotomy with Ureteroneocystostomy, Right Double J Ureteral Stent, Appendectomy, Extensive Lysis of Adhesions - final pathology - borderline serous carcinoma, no invasive implants |
| Nov-Dec 2023 | Extensive hospital stay after surgical intervention for management of multiple thromboemboli and preoperative decompensation |
| 12/27/23 | Patient discharged to rehab center. |
| 1/29/24 | CT angio showing improvement of VTEs |
| 5/10/24 | CT chest, abdomen, pelvis showing no metastatic disease or recurrence |
| 10/10/25 | Normal cardiac stress test performed with cardiology |
| 3/10/25 | Anticoagulation discontinued by hematology |
